# Nanofluid Development Using Silver Nanoparticles and Organic-Luminescent Molecules for Solar-Thermal and Hybrid Photovoltaic-Thermal Applications

**DOI:** 10.3390/nano10061201

**Published:** 2020-06-19

**Authors:** James Walshe, Pauraic Mc Carron, Conor McLoughlin, Sarah McCormack, John Doran, George Amarandei

**Affiliations:** 1 School of Physics & Clinical & Optometric Sciences, Technological University Dublin, City Campus, Kevin Street, D08 NF82 Dublin, Ireland; james.walshe@tudublin.ie (J.W.); c15442598@mytudublin.ie (C.M.); john.doran@tudublin.ie (J.D.); 2 Dublin Energy Lab, Technological University Dublin, City Campus, D08 NF82 Dublin, Ireland; 3 School of Science & Computing, Technological University Dublin, Tallaght Campus, D08 NF82 Dublin, Ireland; pauraic.mccarron@tudublin.ie; 4 The Centre for Biomimetic & Therapeutic Research, Technological University Dublin, City Campus, D08 NF82 Dublin, Ireland; 5 Department of Civil, Structural & Environmental Engineering, Trinity College Dublin, D08 NF82 Dublin, Ireland; mccorms1@tcd.ie

**Keywords:** luminescent down shifting, thermal energy, photovoltaics, fluorescence, spectral beam splitting, nanomaterials, nanofluid, solar energy, photovoltaic-thermal, heat-transfer fluid

## Abstract

Exploiting solar energy using photo-thermal (PT) and/or hybridised photovoltaic/thermal (PVT) systems can represent a viable alternative to the growing demand for renewable energy. For large-scale implementation, such systems require thermal fluids able to enhance the combined conversion efficiency achievable by controlling the ‘thermal’ and ‘electrical’ components of the solar spectrum. Nanofluids are typically employed for these purposes and they should exhibit high heat-transfer capabilities and optical properties tuned towards the peak performance spectral window of the photovoltaic (PV) component. In this work, novel nanofluids, composed of highly luminescent organic molecules and Ag nanoparticles dispersed within a base fluid, were tested for PT and PVT applications. These nanofluids were designed to mimic the behaviour of luminescent down-shifting molecules while offering enhanced thermo-physical characteristics over the host base fluid. The nanofluids’ conversion efficiency was evaluated under a standard AM1.5G weighted solar spectrum. The results revealed that the Ag nanoparticles’ inclusion in the composite fluid has the potential to improve the total solar energy conversion. The nanoparticles’ presence minimizes the losses in the electrical power component of the PVT systems as the thermal conversion increases. The enhanced performances recorded suggest that these nanofluids could represent suitable candidates for solar energy conversion applications.

## 1. Introduction

The process of decarbonizing the European economy, necessitates a reduction in the dependency of European regions on energy derived from fossil fuels whilst simultaneously increasing the percentage of ‘clean’ energy produced [1]. Under different environmental and climate scenarios, the European Union (EU) has committed to having 20% of its total energy consumption generated from renewable sources by the end of 2020, and has planned to increase this percentage to 32% by 2030 [2]. Currently, independently of how the energy is generated (i.e., using fossil fuels or renewables), in industry 70.6% of energy consumption is used for space and industrial process heating, while in EU households heating and hot water alone account for 79% of total final energy use [3]. By 2050, the EU has planned to reach a state of climate neutrality in which 50% of the low- to medium-temperature water (utilized throughout residential, building, and industrial sectors) is delivered by photothermal (PT) technology and 60% of the electricity to be generated by photovoltaics (PV) [1]. However, despite this strong commitment, by the end of 2018, PT systems had accounted for only 7% of the thermal energy that was consumed, while PV systems represent only 4% of the European energy market [2,4]. Consequently, to ensure that solar energy applications become aligned with the legislative changes and to prioritize the integration of solar energy systems, the inherent drawbacks of each standalone technology must be addressed and new technologies should be developed [5]. The most widely recognised route toward achieving these goals has been to improve upon the cost effectiveness of the technology through enhancing its overall energy-conversion efficiency, which, in turn, decreases the energy payback period for the technology as well as ameliorating its carbon footprint [6]. Therefore, by increasing the exergetic efficiency (the amount of useful work that can be extracted) of solar energy technologies (photothermal and photovoltaic), the potential for reaching the energy targets set by the EU could become closer to a reality [1,2,6].

**Figure 1:**
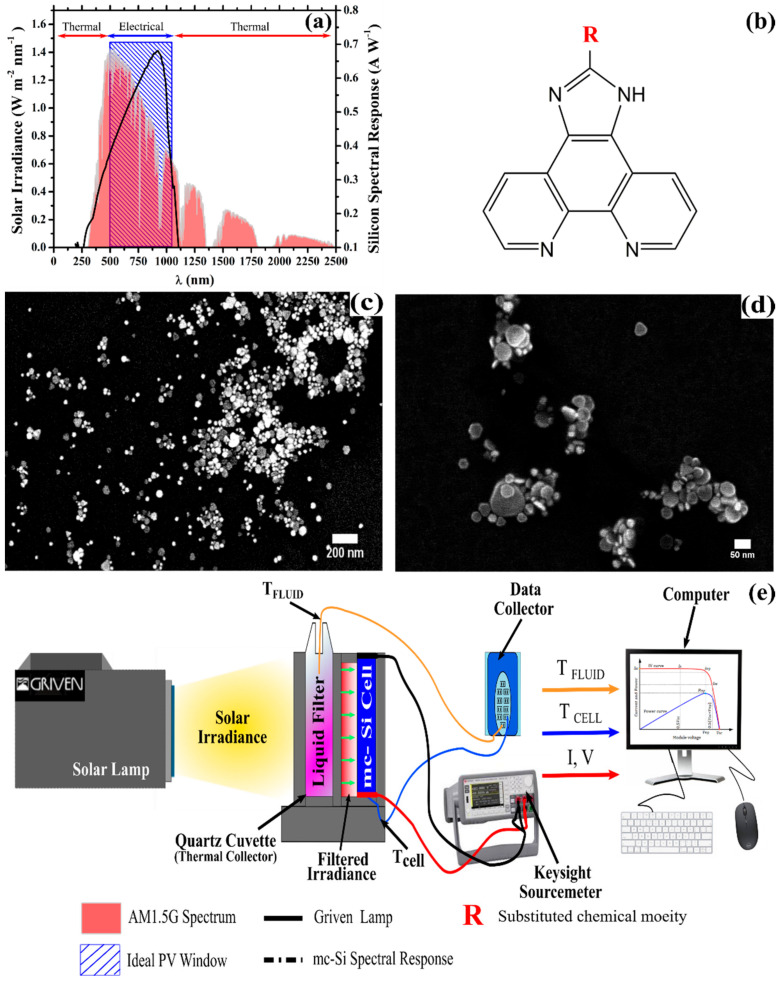
(**a**) Typical solar spectrum and its idealised photothermal (thermal) and photovoltaic (PV) (electricity) conversion spectral regions. The spectral window response of the monocrystalline silicon (mc-Si) and the ideal photovoltaic conversion spectral range for this technology (highlighted in blue) are shown for comparison. Small differences between the two can be observed; (**b**) the general molecular structure underpinning the range of hybridised heat-transfer fluids considered in this study, where R indicates the substitution site for the specific chemical moieties outlined in Table 1; (**c**) scanning electron microscope (SEM) images (scale bar 200 nm) of the silver nanoparticles which contained particle sizes ranging from 10 nm to 85 nm; (**d**) SEM images (scale bar 50 nm) emphasising the extensive range of anisotropic particle morphologies including prisms, hexagons and other non-spherical geometries; (**e**) schematic representation of the experimental set-up used to evaluate the performance of a concentrator PV cell (C-PV) incorporating various liquid plasmonic-enhanced luminescent down-shifting (PLDS) filters designed for concentrated photovoltaic-thermal (C-PVT) systems. The photo-thermal properties were monitored simultaneously.

Despite the prevalent integration of monocrystalline silicon (mc-Si) cells into standalone PV systems and combined heat-power applications, the spectral responsivity of this technology (Figure 1a) continues to remain limited by the fixed energy bandgap of the PV material (see the ideal PV conversion window highlighted in Figure 1a). Consequently, a significant portion of the solar spectrum is either inefficiently converted into usable electricity (fundamental losses in the PV material) or leads to the production of heat [7]. This heat remains completely unexploited in PV applications currently, being commonly either, (i) lost to the environment or (ii) acting as a supplemental limiting factor to the efficiency of the PV systems [8]. One alluring option for circumventing these restrictions is to capitalise on the inherent synergies between PV and PT technologies. This can be achieved by hybridising the two technologies into a single photovoltaic-thermal (PVT) collection system capable of delivering heat and power simultaneously [9,10,11]. Typically, in the majority of the commercial PVT collector designs, the two collection systems (PV and PT) are kept in close physical proximity (with PT being, typically, underneath the PV panel and, consequently, unexposed to the solar radiance) to minimize the additional modifications that were required to the device architecture [9,10,11]. Moreover, in these classical PVT architectures the opportunity for thermal energy to leak from one collection system to the other can obstruct the system’s ability to efficiently convert the solar energy into thermal and electrical power, resulting in lower conversion performances [9,10,11,12].

By thermally decoupling the two-collection systems and employing a careful selection of heat-transfer fluids, further advances in the operating temperature of the thermal component of the PVT systems can be obtained [13,14]. Unfortunately, the typical single-component heat-transfer fluids (e.g., glycols, mineral oils, water etc.), which are frequently employed in the PT or PVT systems, do not yield significant enhancements due to their fixed spectral properties [15,16,17]. In addition, the limited spectral transmittance of these single-component working fluids does not adequately match the spectral responsivity of the commercial PV technology available. This further decreases the potential of the PVT systems to efficiently generate clean electricity when such fluids are used [15,16,17]. Therefore, alongside the developments in PVT architecture, there is the concurrent requirement to identify and/or develop new high-performance heat-transfer fluids whose spectral properties can be controlled, adapted, and optimised for specific solar energy applications. 

A solution to consolidate and augment the recent PVT architecture developments can be the integration of spectral beam-splitting (SBS) technology into the design of PVT devices [18,19]. In this architectural arrangement, up to 95% of the solar irradiance could potentially be captured and utilised [20]. Under laboratory conditions, where the thermal and the electrical behaviour of scaled down SBS-PVT systems are initially evaluated, lower collection efficiencies (~55–83%) are typically achieved due to the stationary nature of the working fluid and the non-optimised geometry of the thermal collection element [21,22]. Thus, the evaluation of the collection systems is typically tested under normal incidence scenarios which also restricts the working fluid temperatures that can be attained. However, these scenarios are reliable tests for characterizing the working fluid properties prior to their testing in large-scale applications [23]. Thus, the working fluid, contained in the thermal collection element, can efficiently filter the solar irradiance (to maximise the production of electricity) whilst also serving as the heat-transfer fluid for the collection of thermal energy [18,19]. In other words, the internal structure of the liquid optical filters acts to partition the solar spectrum into its respective ‘thermal’ and ‘electrical’ components. Consequently, the cooperative interactions between the two collection elements can be maximised [24]. Furthermore, SBS allows for the characteristics of the filter to be readily modified or maintained through the simple replacement of the working fluid [18,25] increasing the cost effectiveness of this strategy when compared to the other alternative hybridisation approaches [11,19]. Under these conditions it is essential to identify materials which not only possess an exceptionally high transmittance over the spectral window where the PV technology is most efficient, but also materials which exhibit exemplary heat-transfer capabilities [24]. Hence, the desire to produce and evaluate the optical and thermophysical properties of different combinations of material additives (e.g., Ag, Cu, Au, carbon, oxides nanoparticles (NPs) etc.) which have been suspended in a base fluid (such as water, ethylene glycol or other single-component heat-transfer fluids) has become an area of significant research interest, leading to the development of various types of nanofluids [15,22,26,27]. 

Traditionally, the material strategies that have been employed to promote the enhancement of SBS-PVT performance have been centralised around the dispersal of a single type of nanomaterial (metals, polymers, ceramics, or carbon-based particles etc.) throughout a base fluid [23,27,28,29,30]. The ability to control the optical and thermophysical properties of these nanofluids can be improved by the modification in the particle shape, size, and size-distribution of the NPs dispersed within the base fluid. Such approaches demonstrated considerable enhancements in the collective efficiency of the liquid optical filters when implemented in SBS-PVT [13,21,23,26,31] or, separately, in PT systems [30,32,33,34]. For example, in SBS-PVT systems the implementation of Ag/TiO_2_ and Ag-SiO_2_ nanoparticles has enabled combined conversion efficiencies of ~83% and ~55% to be achieved, respectively [21,22]. However, in each configuration the proportion of the solar irradiance that was converted to clean electricity was reduced to less than 10% [21,22]. As a result, the subtle differences between the exergetic quality of the thermal energy and the electrical energy (electricity) needs to be considered when examining the performance enhancements reported for SBS-PVT systems [23]. Electrical energy is defined as 100% recoverable work (having exergy equal to its energy), while thermal energy is defined as low-grade exergy due to the difficulty of extracting its energy content (exergy < energy) [23]. In non-concentrated SBS-PVT systems encountered in low- to moderate-temperature residential and industrial applications, in the promise of generating additional low-grade thermal energy, the exergetic loss of ‘clean’ electrical energy (due to the losses experienced in PV performance) can negatively impact the payback period, the carbon footprint, and the benefits of the hybridised architecture [23].

For these nanofluids to become a suitable alternative to the more cost-effective single-component heat-transfer fluids (that are already commercially available), a number of limitations in their design will need to be overcome [30,35,36,37]. First it is the prerequisite requirement of the often-expensive precursor materials [38], some of which are known to be environmentally hazardous and can cause adverse health effects [39]. Secondly, it is the inability of nanofluids to maintain their internal configuration and, hence, their heightened heat-transfer capability after prolonged periods of exposure to the high intensity irradiance and at the temperatures required in SBS-PVT applications [39]. Finally, it is the lack of diversity in the nanofluid filter designs in which the nanoparticles, typically, have a monomodal particle size-distribution or exhibit a single particle morphology [35,36,37,38]. This lack of diversity in the complexity of the nanoparticle population has now been widely recognised to constrain the enhancements reported in the thermal conductivity and the heat-transfer dynamics of the nanofluids currently implemented in PT and PVT devices [40,41]. Recently, the presence of a large ensemble of different particle-sizes and the inclusion of different anisotropic particle morphologies has shown to further promote the transport of thermal energy throughout the base fluid, yet the cross-application of this aspect to SBS-PVT systems remains largely unexploited [34,42,43,44,45].

In previous designs, the integration of organic and organometallic fluorescent molecules was investigated by our group in developing a luminescent down-shifting (LDS) strategy for PV applications [46], while Ag-based nanofluids were successfully tested for PT systems [33]. The positive results obtained suggested that such compounds (containing fluorescent molecules and/or Ag nanoparticles) could be also be implemented as liquid SBS layers for PT and/or PVT applications. In this scenario the new working fluids could simultaneously enhance the spectral selectivity of the base fluid and its heat-transfer capabilities, whilst also exhibiting a controllable fluorescence emission in the optimal spectral conversion window of the PV element [46]. Consequently, when employed in the PVT systems, the LDS working fluids can potentially minimize the loss of electrical energy that often occurs through the inclusion of the thermal component in the classical hybridisation process [27,31]. However, rather than competing, the two distinct strategies (LDS and nanofluids) can be consolidated into a single working fluid in which the presence of the nanoparticles not only enhances the thermal contributions, but also promotes the intensity of the fluorescence in the down-shifting species [47]. The fluorescent behaviour of a molecule is typically modified by the presence of the highly localised electromagnetic fields that are established on the surface of metal nanostructures as a result of localised surface plasmon resonance (LSPR) [47]. In addition, the interactions between neighbouring nanoparticles of different particle sizes and different particle shapes within a network of particles has already been shown to further enhance the heat-transfer dynamics of the base fluid in which they are suspended [35,39]. Thus, the concentration of the nanoparticles can be adjusted to tune the interactions of the incoming solar flux with both the base fluid and the luminophore to further enhance the capability of the filter to modulate between the thermal (non-radiative contributions) and electrical (radiative contributions) outputs. This complimentary capacity to fine-tune the light–matter interactions (absorption, fluorescence, and scattering) within the liquid filter could further ameliorate the electrical energy lost through the inclusion of the thermal component within concentrated PVT (C-PVT) systems. Furthermore, this could increase the thermal performances achievable with conventional nanofluid-based approaches. Therefore, integrating such a plasmonic-enhanced luminescent down-shifting (PLDS) nanofluid in SBS-PVT applications may potentially further compensate for and overcome the losses typically encountered in the hybridisation processes. Thus, this approach has the potential to facilitate higher solar energy-conversion efficiencies. 

In this study, to recover the ‘waste’ heat generated through the spectral mismatch between the incident solar irradiance and the conversion dynamics of the PV material and, also, to enhance the combined conversion efficiency of C-PVT systems, spectrally selective PLDS nanofluids were designed to target the ‘thermal region’ whilst simultaneously maintaining/enhancing the efficiency of the ‘electrical region’, shown in Figure 1a. The internal structure of these fluids consisted of a subset of the imidazo[4,5-*f*][1,10]phenanthroline (Imphen) derivatives, previously developed and explored by our group [46], and silver nanoparticles which were fabricated using a two-step wet chemical synthesis process which is based on abundantly available and relatively low-cost reagents [34]. The combination of these two approaches to create PLDS nanofluids facilitates the capture and the conversion of high-energy photons from within the 300–450 nm ‘thermal region’ into radiation with wavelengths that are more energetically beneficial and utilisable by the PV material. At the same time, this combined approach also enables a greater degree of control over the optical and thermophysical behaviour of the working fluids. In this work, the objective was also to identify if the presence of the nanoparticles and their complex ensembles could assist in further promoting the fluorescent contributions from the down-shifting molecules and/or enhance the thermal properties of the base fluid (here, ethylene glycol) in which the two species were dispersed. The interaction between the two species (fluorophore, nanoparticle) was evaluated through the changes registered in the working fluids absorption and emission bands, the optical transmittance, the heat-transfer dynamics in PT systems and the consolidated effect on maximising the collective (thermal and electrical) power efficiency of a hybrid SBS-PVT system. In addition, the economic viability of the proposed modifications to the LDS fluids (and, consequently, the SBS-PVT configurations envisioned) was ascertained through implementing a merit function which was calculated using the most up to date information for Ireland and the European region [48,49].

## 2. Materials and Methods 

### 2.1. Materials 

A monocrystalline silicon (mc-Si) cell manufactured by Big Sun Community Solar (San Antonio, TX, USA) was selected for this study based on the widespread inclusion of this PV technology in the majority of the low to medium C-PVT systems [24]. To limit the fundamental losses of this PVT system, the luminescent down-shifting materials were integrated into the filters design through the implementation of highly fluorescent organic ligands. The structure of the fluorescent ligands consisted of a 1*H*-imidazo[4,5-*f*][1,10]phenanthroline core which was functionalized at the R-position designated in Figure 1b with one of three distinct chemical moieties. The 1*H*-imidazo[4,5-*f*][1,10]phenanthroline derivatives incorporated here contain the key functional groups which were identified to enhance the fluorescent character of the molecules. These molecules were selected for this specific application from a larger study, conducted by our group, onto the relationship between the functional component utilised and the fluorescent properties of the resulting ligand [46]. A full description of the molecules that were synthesised and investigated is presented in Table 1 together with their respective formula, nomenclature, molecular weight, and their abbreviation (which will be adopted throughout the manuscript).

### 2.2. Plasmonic-Enhanced Luminescent Down-Shifting (PLDS) Nanofluid Preparation

The PLDS fluids outlined in Table 1 were prepared via a two-step process. Firstly, silver nanoparticles with an average particle diameter of 10–85 nm were synthesized using the protocol previously explored by our group for standalone solar thermal (PT) applications (for further details see [34] where the complete synthesis procedure is described). All of the reagents were purchased from Sigma Aldrich (Sigma Aldrich, St. Louis, Missouri, United States) and used without further purification. This nanosynthesis protocol was selected based on its ability to produce a broad distribution of particle sizes (Figure 1c) and morphologies (Figure 1d) [34]. In short, in a such synthetic procedure, ‘seed’ nanoparticles (2–10 nm in diameter) were first synthesised through the reduction of silver nitrate (2 mL 0.001 M) in the presence of polyvinyl alcohol (2 mL 1% *w*/*v*) via sodium borohydride (2 mL 0.001 M) [34]. From this ‘seed’ solution 1 mL was used to control the particle growth which was supported by the presence of hydrazine (5 mL 0.1 M), tri-sodium citrate (3 mL 0.1 M), and polyvinyl alcohol (1 mL 1% *w*/*v*)—which collectively formed the ‘growth medium’ [34]. Finally, 4 mL of silver nitrate (0.001 M) was added in a dropwise manner to the ‘growth medium’ to form nanoparticles of the desired size-range [34]. The colloidal suspension which resulted (~12 mL) was subsequently centrifuged at 10,000 rpm for 45 min before the supernatant was removed and the remaining nanoparticles were transferred into 3 mL of pristine ethylene glycol. This processing stage ensured that the effects of the unwanted chemical species (unreacted/reactive ions), often present in colloidal dispersions, had minimal influence on the optical and thermal response of the working fluids towards the solar flux.

A highly concentrated stock solution of each fluorescent compound was prepared by dissolving ~0.03 g of each compound in 2.5 mL of pristine ethylene glycol. The fluorescent solutions were subjected to one minute of mechanical agitation using a VWR vortex shaker (VWR International Ltd. Dublin, Ireland), before being transferred to a ULTRAsonik 57X ultrasonication bath (Ney Dental Inc. Braunschweig, Germany) for 1 h of sonication. The combination of both mixing stages ensured that the fluorescent compounds were homogenously dispersed throughout the ethylene glycol prior to their utilization in the formation of the PLDS working nanofluids.

The PLDS working nanofluids, which will act as liquid optical filters, were prepared by taking aliquots from each stock solution (fluorescent material, nanoparticles) and mixing them accordingly. Each working fluid consisted of a fixed concentration (0.005 wt%) of the fluorescent material (P282, P205 or P183) and contained either a 1%, 5% or 10% *v*/*v* concentration of the nanoparticle solution. In addition, the particle size of the LDS and PLDS working fluids was measured and analysed (see Supplementary Materials) using a Malvern Nano series ZS Zetasizer (Malvern Instruments Ltd., Malvern, UK). Hydrodynamic particle size measurements were performed in quartz glass cuvettes under ambient condition using a He-Ne Laser (λ = 633 nm) at detector angle of 173°. This quality control step ensured that any aggregates present in the working fluid solutions were recorded prior to their exposure to the intense solar irradiance and elevated temperatures encountered in evaluation of the fluids in Section 2.3.3.

For the new PLDS nanofluids considered in this study, the cost vs. performance enhancement represented a core part of the initial synthetic design. Therefore, the choice to utilize the 1*H*-imidazo[4,5-*f*][1,10]phenanthroline LDS molecules and silver based nanoparticles to generate the PLDS nanofluids was carefully considered, as were their synthetic protocols. The LDS molecules are based on the 1,10-phenanthroline core structure and have been synthesised using an optimized, novel, cost effective and high-yielding synthetic route. The cost per gram to produce each of the Imphen LDS molecules studied (P183, P205, P282 in Table 1) has been estimated to be between 0.90–1.10 € per gram of pure product. The maximum loading of the LDS molecules in each of the working PLDS nanofluids tested was 0.005 wt% (approximately 56 µg of LDS material per litre of ethylene glycol) which equates to additional cost of between 5.00–6.13 cent per litre of LDS loaded ethylene glycol. 

The toxicity and environmental impact of these novel first-generation PLDS nanofluids was also considered at this initial stage. The toxicity of the 1,10-phenanthroline based Imphen LDS molecules and their associated metal complexes has previously been assessed in vitro using numerous mammalian cell lines (VERO, HaCAT) and also using an in vivo *Galleria mellonella* insect model (which is comparable to those derived from murine studies). Thus, it was found that these molecules seems to be highly tolerated and essentially non-toxic at the concentrations utilised in the PLDS nanofluids [50,51,52,53] However, additional and more in-depth toxicity/environmental impact studies on the working PLDS nanofluids will have to be conducted in order for these molecules to be implemented, but such studies will form the basis of future investigations.

### 2.3. PLDS Nanofluid Characterisation

#### 2.3.1. Optical Transmittance of PLDS Filters 

The optical transmittance of the working fluids is the most important measured parameter of the PLDS filters as it directly controls the thermal and the electrical behaviour of the PVT system in the model outlined in Section 2.4 [31]. The optical transmittance spectra were recorded over the 250–2000 nm spectral range using a Perkin Elmer Lambda 900 U*v*/*v*IS/NIR spectrometer (Perkin Elmer Inc. Massachusetts, USA) and having air as a reference [31]. The experimental spectra were acquired under ambient conditions using highly transparent 2 mm path-length quartz cuvettes. The 1 nm wavelength scanning intervals were used to acquire the spectra using the spectrometer. The measurements were repeated 3 times, the average values being those presented. The transmittance spectra recorded from a single path-length measurement can contain unaccounted absorptive (within the cuvette) and reflective (at each optical interface) interactions which could artificially influence the thermal, electrical, and economic behaviour of the PLDS filters when examined under the modelling scenario (Section 2.4). Therefore, the utilisation of multiple path-lengths is essential to measure the value of the fluid’s transmittance at a particular wavelength without the presence of these unwanted optical and reflective losses. Consequently, for each liquid considered, a combination of 2 mm and 5 mm optical path-lengths was used, all measurements being performed twice. The average value was used in all subsequent calculations in Section 2.4. In addition, the calculated transmittance spectra do not include either the fluorescent contributions from the materials or the enhanced fluorescent contributions which arise through the inclusion of the silver nanoparticles. Consequently, the divergences between the experimental spectra (recorded at a single path-length) and the calculated spectra in the absence of optical losses (utilised in the model) were exploited to analyse the influence of the fluorescence and plasmonic contributions to the short-circuit current density delivered by the PV cell in Section 2.3.3. This comparison does not allow the separation of the individual contributions from each species, but it facilitates the quantification of the capability of the PLDS fluids to address some of the loss mechanisms frequently encountered in PVT systems.

#### 2.3.2. Fluorescence of PLDS Filters 

Unlike conventional heat-transfer fluids, the PLDS working nanofluids considered here exhibit a complimentary fluorescent capability and, consequently, their fluorescent spectra had to be analysed. In addition, these measurements also helped to identify the promotion or reduction of the radiative transitions within the fluorescent compounds in the presence of the silver nanoparticles. Thereby, the concentration-dependent effects of the nanoparticles were monitored and any effect they have on the interaction between the two species was investigated. The emission spectra of the PLDS filters were recorded using a Perkin Elmer LS55 luminescent spectrometer (Perkin Elmer Inc. Massachusetts, USA) and 2 mm path-length quartz cuvettes. This optical path-length was chosen as it represented the experimental conditions for which the filters would be employed when recording the current–voltage (I–V) curves under the solar irradiance (see Section 2.3.3). An excitation wavelength of 380 ± 10 nm was selected as it corresponded to a relatively flat spectral window of low absorptivity for the assortment of fluorescent compounds investigated. This decision facilitates the minimization of any inner-filter effects that are frequently known to occur when exciting a fluorescent molecule at or close to its peak absorption wavelength [54]. All spectra were recorded under ambient conditions and employed the same instrument parameters (excitation slit width, photomultiplier voltage, emission slit width etc.), which enabled a comparison across the range of materials.

#### 2.3.3. Performance Evaluation of PLDS Liquid Filters 

The photothermal properties of the PLDS nanofluids and their impact on the electrical characteristics of the concentrator PV (C-PV) silicon cell (25 mm × 25 mm) was investigated using the experimental set-up presented in Figure 1e (complimentary schematic provided in Figure S1). In a typical measurement, the mc-Si PV cell was secured inside a 3D printed polylactic acid (PLA) holder which facilitated the control (through a window of 7 mm × 25 mm) of the spectral irradiance transmitted to the solar cell. This experimental configuration removed all of the unwanted background light contributions to the spectral irradiance and minimised the loss of ‘down-shifted’ or ‘scattered’ photons resulting from the light–matter interactions with the PLDS filters. Once secured inside the holder the mc-Si cell was maintained in the same position. Consequently, the same area of the PV material (7 mm × 25 mm) was exposed to the incoming irradiance during the experiment. Under the 1000 W m^−2^ irradiance provided by the solar simulator (Griven 1200 MSR, Griven LTD, Castel Goffredo, Mantova, Italy), the I–V response curve of the mc-Si C-PV cell without any liquid filter (the ‘unfiltered’ case) was first recorded using a Keysight (Keysight Technologies, Santa Rosa, CA, USA) B2901A source meter. The primary electrical characteristics of the mc-Si C-PV cell including short-circuit current density (J_SC_), open circuit voltage (V_OC_), fill factor (FF), and conversion efficiency (η) were derived from the I–V curves generated at 5 min intervals throughout the 60 min exposure cycles. The electrical measurements for each configuration of the liquid filters was independently repeated in triplicate, with the average values being presented in the discussions. Additionally, the PV cell was prevented from being actively cooled throughout the exposure period to enable any partial shading capabilities that arose as a result of the internal configuration of the PLDS filters to be investigated. Accordingly, the temperature of the silicon cell (T_CELL_) was monitored throughout the exposure period (60 min) using a K-type thermocouple (±0.05 °C) to ensure that any differences in the electrical performance of the PV cell could be adequately compared across the range of liquid filters investigated. 

Subsequently, a 2 mm path-length quartz glass cuvette (L × W × H: 120 mm × 2 mm × 75 mm) was placed inside the 3D printed PLA holder whose design also minimised the thermal losses through the cuvette walls and acted as an insulator. The quartz glass cuvette was filled with 1 mL of a nanofluid (containing a fluorophore and silver nanoparticles) and then placed between the PV cell and the solar lamp as shown in Figure 1e. An additional K-type thermocouple was placed into the quartz glass cuvette to monitor the working fluid’s temperature (T_FLUID_) at 1-minute intervals throughout the exposure period. This temperature data was subsequently used to calculate the nanofluids photothermal conversion efficiency according to the methodology outlined in Section 2.3.4. After each exposure cycle had been completed, a cooling period of 20 to 30 min was allocated to ensure the PV cell and fluid temperatures returned to the initial temperature value. In addition, the particle size of the LDS and PLDS working fluids post-exposure was measured and analysed (see Supplementary Materials) using a Malvern Nano series ZS Zetasizer (Malvern Instruments Ltd., Malvern, UK). This analysis ensured that any modifications in the internal structure of the working fluid solutions, due to exposure to the intense solar irradiance and elevated temperatures, were recorded. While this type of analysis does not quantify the capability of a particular fluid to maintain its enhanced performance after repeated exposures cycles, it does enable a comparison to be drawn in terms of how the materials are behaving in response to the irradiance and thermal conditions encountered in the PVT application. In this manner, the stability of the working fluid solutions in the presence of the nanoparticles and the impact of the nanoparticle concentration can also be assessed.

The I–V curves of the filtered devices are, in these experimental configurations, influenced by the interaction of the solar irradiance with the housing of the thermal collector (the quartz cuvette), by the reflection losses arising from the air/liquid/quartz interfaces and by the incidence of plasmon assisted fluorescent contributions from the materials considered. Consequently, the differences between the J_SC_ acquired under these experimental conditions and J_SC_ determined under the modelling scenario detailed in Section 2.4 allowed the investigation of the capability of the fluorescent contributions (in the presence of silver nanoparticles) to overcome some of the loses encountered in hybridisation.

#### 2.3.4. Photo-Thermal Conversion Efficiency of PLDS Filters

Alongside the investigation into the behaviour of the electrical component with the addition of the PLDS filters in Section 2.3.3, it remained essential to determine the accompanying changes registered in the performance of the thermal collection unit. The photothermal conversion efficiency (PTE) of a heat-transfer fluid is commonly defined as the ratio between the energy stored within the heat-transfer fluid (in this case the PLDS filters) and the total incident solar irradiation [34]:

(1) $$PTE=\frac{\left(c_{1}m_{1}\Delta T_{1}+c_{2}m_{2}\Delta T_{2}\right)}{IA\Delta t}\approx \frac{c_{1}m_{1}}{IA}\left(\frac{\Delta T_{1+2}}{\Delta t}\right)$$

where *c_1_* and *c_2_* are the specific heat capacity, *m_1_* and *m_2_* are the mass, *∆T_1_* and *∆T_2_* are the instantaneous increase in the temperature over the measurement period Δt of the base fluid (1) and the nanoscale additives added to the fluid (2), respectively; *I* is the irradiance and *A* is the illumination of the heat-transfer fluid. Under the non-concentrated low-intensity conditions, the temperature difference between the additives and the base fluid is negligible [34]. The low concentrations of the molecular additives employed allows their mass contribution to be assumed as negligible [34]. This allows Equation (1) to be simplified, with the PTE being now directly proportional to the temperature gradient at a point within the bulk heat-transfer fluid ΔT_1+2_. This was measured by the thermocouple inserted in the fluid in 2.3.3. This approach was utilized as an efficient method for identifying the combinations of the working fluid constituents (nanoparticle and LDS materials) which resulted in the highest PVT conversion performances, and removed the need for measuring the thermophysical parameters of each individual configuration of the working fluid. However, a complete fundamental study of a newly developed material’s thermal properties is required to be considered for a real-world thermal application. Such a study should look into the influence of various concentrations (both from fluorescent molecules and Ag nanoparticles perspectives) on the thermal properties, fluid viscosity etc. in order to optimize the fluid characteristics. Therefore, such a study remains outside the scope of the present work. The enhancement in the PTE of the modified heat-transfer fluid, as compared to the base fluid, offers a clearer metric to identify the impacts of specific design parameters on the performance of the fluid within a given system.

(2) $$PTE\;Enhancement=\left(\frac{PTE_{PLDS}-PTE_{base\;fluid}}{PTE_{base\;fluid}}\right)\times 100$$

### 2.4. Merit Function of Spectral Beam-Splitting Photovoltaic-Thermal (SBS-PVT) System with PLDS Liquid Optical Filters

Although the tests outlined in 2.3.3 and 2.3.4 adequately allows to quantify the impact of a particular liquid filter on the conversion performance metrics of the C-PV silicon cell and for the accompanying thermal collector to be evaluated under differential temperature loads, it cannot distinguish the energetic and economic dynamics of the thermal and electrical collector performances. Therefore, a theoretical approach was additionally employed to reveal the combined power output (thermal and electrical) delivered by each liquid PLDS filter when it is utilised in a C-PVT system. The ‘unfiltered’ and ‘filtered’ performances of the silicon C-PV cell and the accompanying thermal collection unit were modelled according to the previous method detailed in [31]. In short, the electrical power generated by the C-PV mc-Si cell (P_PV_) when filtered by the various PLDS nanofluid filters prepared in 2.2 was evaluated using the product of the PV cells fill factor (FF), open-circuit voltage (V_OC_)and the short-circuit current density (J_SC_) delivered under the filtered irradiance [31]:

(3) $$P_{PV}=V_{OC}*FF*J_{SC}\left(filtered\right)=V_{OC}FF\int_{250\;\mathrm{nm}}^{2000\;\mathrm{nm}}\phi_{AM1.5D}\left(\lambda \right)\cdot SR\left(\lambda \right)\cdot T_{Liquid}\left(\lambda \right)\cdot d\lambda$$

where $\phi_{AM1.5D}$ is the photon flux contained in the standardised AM1.5G solar spectrum, SR is the spectral response of the C-PV cell utilised, $T_{Liquid}$ is the optical transmittance of the filter in the absence of the interactions with the glass/air/liquid interfaces (i.e., the transmittance spectra calculated using the different path lengths in 2.3.1), and $V_{OC}$ and FF are determined through a series of equations which dictate the electrical behaviour of a PV cell [31]. Alongside the electrical power delivered by the SBS-PVT system, the generated thermal power (P_TH_) is derived using Equation (4) which is based on the thermodynamics of the collector design adopted in the model’s formalism [31]:

(4) $$P_{TH}=\eta_{collector}\int_{250\;\mathrm{nm}}^{2000\;nn}\phi_{AM1.5D}\left(\lambda \right)\cdot \left[1-T_{Liquid}\left(\lambda \right)\right]\cdot d\lambda$$

where $\phi_{AM1.5D}$ is again the photon flux of the AM1.5D solar spectrum, $1-T_{Liquid}$ is the energy absorbed by the fluid as a result of its internal structure, and $\eta_{collector}$ is the thermodynamic limit of efficiency for the energy collection process [55]. Consequently, the efficiency ($\eta_{*}$) of the electrical or thermal element can be calculated by:

(5) $$\eta_{*}=\frac{P_{*}}{\int_{250\;\mathrm{nm}}^{2000\;\mathrm{nm}}\phi_{AM1.5D}\left(\lambda \right)\cdot d\lambda }$$

where $P_{*}$ can be substituted with the $P_{PV}$ or $P_{TH}$ values. The denominator in Equation (5) is simply the power available in the AM1.5D solar spectrum. The capability of the PLDS working fluids to effectively convert the solar energy into thermal and electrical energy within C-PVT systems was ascertained through the implementation of a merit function (MF):

(6) $$MF=\frac{w*P_{PV}+P_{TH}}{w*P_{PV}\left(unfiltered\right)}$$

where $P_{PV}$ and $P_{TH}$ designate the electrical power and thermal power, respectively, w is the worth factor (ratio of the cost of thermal energy to electricity in currency/kilowatt hour), and $P_{PV}(unfiltered$) is the electrical power delivered by the standalone PV system. A full description of the calculations involved in this quantity and its significance in assisting in the design and evaluation of heat-transfer fluids for PVT systems is detailed in [21,31]. In this analysis context, a merit function value of 1 implies that the hybridisation of the two collection elements (the ‘filtered’ PV and PT systems) has resulted in an economically and energetically favourable configuration of the PVT system operating under the specific economic/geographical considerations imposed by the model [56]. Consequently, any subsequent improvements in the value of the merit function as a result of modifying the spectral properties of the working (by the LDS material(s) and/or the nanoparticles) will represent further enhancements in the economic and energetic performance of the SBS-PVT system [56]. Therefore, analysing the value of the MF in response to the optical properties of the LDS materials, the concentration of the NPs, and the economic considerations (as imposed by a specific region of envisioned employment of the SBS-PVT systems) allows for the performance of the collection system to be readily optimised.

The results of the merit function analysis presented here were determined using 2018 economic data from across the European region [48,49], which lead to a worth factor (w) value of 3.09 ± 0.89. This approach allowed for the fluctuations in the energetic and economic merit of the PLDS working fluids to be investigated across a range of different economic climates. Additionally, this approach permits for ‘geographical screening’ of the working fluids, helping to identify suitable locations where their evaluation can be scaled up and their behaviour under experimental conditions could be realised. This can also allow for the individual molecular structures characterised to be uniquely tailored towards deployment in specific geographical locations, in order to maximise their combinatory conversion efficiency when deployed within a PVT system.

## 3. Results and Discussion

### 3.1. Optical Transmittance and Fluorescence of PLDS Nanofluid Filters

The spectral deviations in the optical transmittance of the nanofluids working as liquid optical filters in each of the partitioned spectral regions (outlined in Figure 1a) were monitored. The solar irradiance available (Figure 1a) is ideally separated into the following spectrally-distinct energy conversion regions (1) thermal energy (from 250–500 nm and from 1100–2000 nm) and (2) electricity (from 500–1100 nm). Therefore, the spectral analysis of the proposed nanofluids in response to the different nanoparticle concentrations will be examined and discussed in relation to these distinct energy regions.

**Figure 2:**
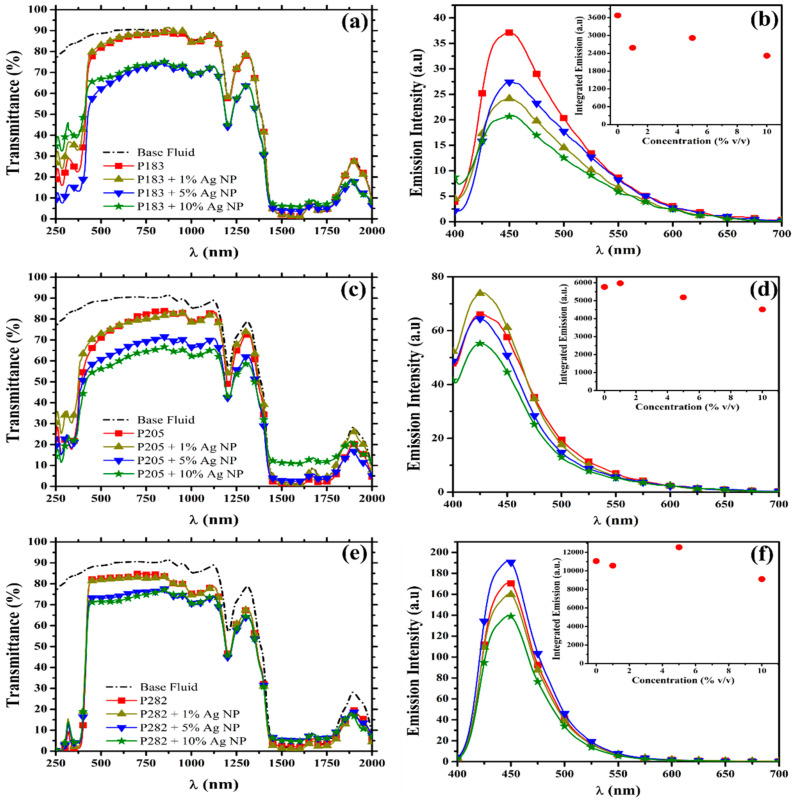
Experimentally determined optical transmittance of (**a**) P183, (**c**) P205 and (**e**) P282 loaded ethylene glycol based PLDS nanofluids at different Ag nanoparticles concentration. The transmittance of the base fluid is included for comparison. Their corresponding fluorescent intensity as a function of the loading concentration of nanoparticles are presented in (**b**), (**d**), and (**f**), respectively. The integrated emission intensity as a function of the nanoparticle concentration is provided in the insets for comparison.

The experimentally measured optical transmittance spectra of all three types of the PLDS nanofluid filters listed in Table 1 are presented in Figure 2 (P183-a, P205-c, P282-e), with the accompanying spectral properties of the base fluid provided for comparison. This figure in combination with the modelled transmittance spectra for each configuration of the nanofluid (presented in Figure S2 in the Supplementary Materials) allowed for an in-depth analysis of the interaction of the solar flux with the liquid optical filters. The spectral region in which each type of fluorescent material is known to be optically active is the 250–500 nm thermal region, while the optical activity for the particular size-distribution of nanoparticles employed is known to extend from 25–600 nm [34,46]. This aspect has been previously confirmed through a series of independent measurements of each species absorption band at very low concentrations [34]. Indeed, each one of these signature spectral contributions can be observed in the transmittance spectra of the nanofluids in Figure 2 when examined under the increasing nanoparticle concentration.

In the absence of any nanoparticles, the purely organic luminescent down-shifting species enhanced the absorption of the base fluid by 50% (P205—Figure 2c) to 61% (P282—Figure 2e) within the 250–500 nm thermal spectral region. In addition, a marked increased absorption of 2% (P183—Figure 2a) to 20% (P282—Figure 2e) in the 1100–2000 nm thermal region was also observed. The enhanced absorption at such large-wavelengths is likely the result of the scattering processes which can appear due to the interactions of the solar flux with the large aggregates (diameter ~80–5500 nm). These aggregates can be seen in the particle-size data for the pristine pre-exposed LDS fluids (Figure S3). In contrast, 3% (P183—Figure 2a) to 8% (P205—Figure 2c) of the energy available for conversion into electricity by the PV component was lost due to the dispersion of the organic materials throughout the base fluid. This loss in the transparency of the working fluid solutions could be in part due to the intrinsic (and somewhat limited) solubility of the luminescent materials in the base fluid (ethylene glycol) or could be the result of the scattering contributions which originate from the smaller sized aggregate species present.

As nanoparticles begin to be introduced into the hybridized working fluids at low concentrations of 1% *v*/*v*, an initial 7% (P183—Figure 2a)–9% (P205—Figure 2c) reduction in the absorption within the high-energy thermal region (250–500 nm) appears in the spectra. At the same nanoparticle concentration, some small variations (−9% to +2%) in the transmittance of the nanofluids in the 1100–2000 nm thermal spectral region can also be observed (P205—Figure 2c, P282—Figure 2e). Furthermore, few additional spectral deviations were observed in the transparency of the nanofluid solution across the electricity region (500–1100 nm) with the introduction of this small concentration (1% *v*/*v*) of nanoparticles. As the concentration of nanoparticles is further increased the possibility of the interaction between neighboring nanoparticles and/or the physical and the chemical interactions between the two species (nanoparticles and Imphen molecules) becomes intensified. The combined effect of these interactions is exhibited by the emergence of a non-linear dependence in the variations of the transmittance spectra of the nanofluids with increasing nanoparticle concentration. In most instances (with the exception of P183), the inclusion of the nanoparticles in the PLDS nanofluid contributed to a 5% to 17% (250–500 nm) and 3% to 20% (1100–2000 nm) enhancement in the absorption of the designated thermal regions respectively, when compared to the pristine LDS fluid configurations. Consequentially, it is expected that the integration of these PLDS nanofluids in SBS-PVT systems will contribute to the production of more thermal energy at the modest expense of some small losses in the electrical efficiency of the PV component.

In addition to the enhanced absorption capabilities exhibited by the PLDS working fluids in the targeted thermal regions, the fluorescent contributions within the electricity spectral region in the presence of the nanoparticles was also considered in the evaluation of the PLDS nanofluids. In the pristine LDS configuration (i.e., in the absence of the nanoparticles) the fluorescent intensity of the organic working fluids follows the order of successively decreasing magnitude: P282, P205, followed by P183. For comparison, the fluorescent intensity exhibited by the P282 filter (Figure 1f—red square) is 3 times greater than the fluorescence exhibited by the P205 configuration (Figure 1d—red square), whose emission is correspondingly 1.5 times greater than the fluorescence contributions delivered by the P183 fluid (Figure 2b—red square).

As the nanoparticles become integrated into the internal structure of the liquid optical filters, the fluorescent behavior of each luminescent species becomes divergently modified (Figure 2). This divergence in the fluorescent behavior of the different luminescent materials is exemplified when comparing the responses of P183 (Figure 2b—yellow triangle) and P205 (Figure 2d—yellow triangle) to the inclusion of 1% *v*/*v* concentration of nanoparticles. In the case of P183 the nanoparticles contributed to a ~30% reduction in the fluorescent intensity, while for the P205 species the nanoparticles enhanced the emission intensity by ~3%. This optical behavior in the presence of the nanoparticles is not unexpected as the modifications introduced into the luminescent quantum yield of highly emissive species (like the molecules considered here) is often found to be less significant than the enhancements achieved with less efficient emitters.

As the concentration of the nanoparticles in the liquid filters is increased, the interparticle distance between the luminescent species and the nanoparticles decreases [47]. The resulting close proximity of the two species gives rise to a higher probability of the incoming solar irradiance to be directly absorbed by the nanoparticles rather than the energy being internally transferred into the luminescent material for radiative re-emission [47]. This transition in the behavior of the nanofluids is confirmed by the 18% (P282—Figure 2f) to 37% (P183—Figure 2b) decrease in the fluorescent intensity of the filters with a 10% *v*/*v* nanoparticle concentration (Figure 2—green star). Consequently, the nanoparticles, in these instances, are absorbing more of the solar energy through non-radiative processes which leads to a decreased fluorescent intensity witnessed in the emission spectra of the filters (Figure 2). Furthermore, none of the tested nanoparticle concentrations was found to unanimously maximize the cooperative interactions between the two species. The optimal concentration for maximizing the fluorescent intensity seems to be designated on a material by material basis and, hence, on molecular structure basis (Figure 2). The optimal concentration identified for each type of PLDS filter was 0% *v*/*v* for P183, 1% *v*/*v* for P205 and 5% for P282. However, considering the nanoparticle concentration-dependent effects on the nanofluid’s optical transparency, absorption, fluorescence, and plasmon-enhanced fluorescence, the optimal concentration which maximizes the energetic and economic merit of the PVT system may not have been captured in this study and requires further detailed investigation.

### 3.2. Stagnation Temperature and Photothermal Conversion Efficiency (PTE) of PLDS Nanofluid Filters

**Figure 3:**
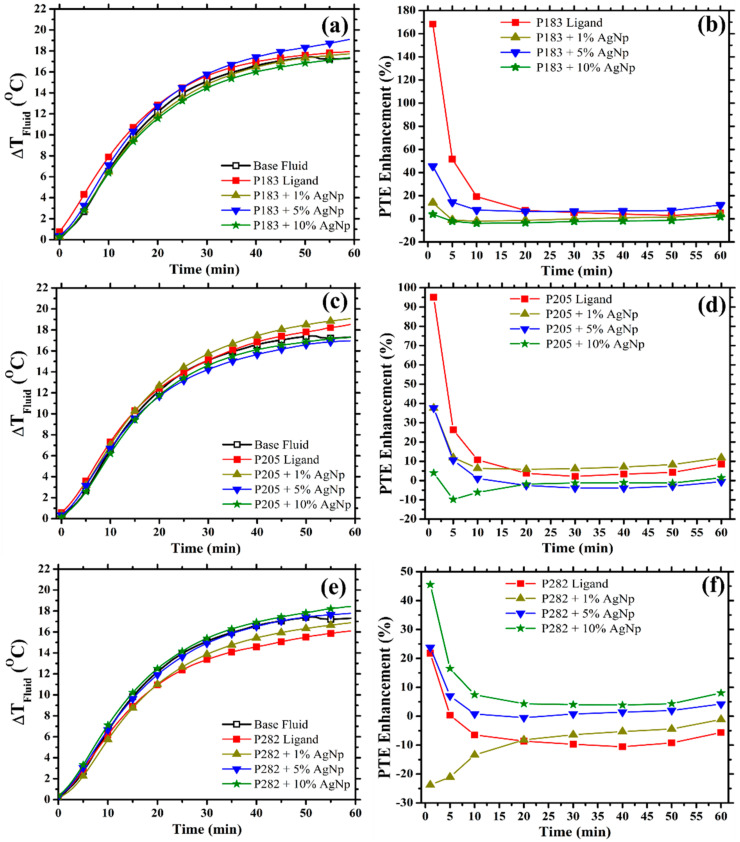
Variation in the temperature response ($\Delta T_{Fluid}$) of the PLDS nanolfuid filters ((**a**)—P183, (**c**)—P205, (**e**)—P282) and the accompanying change in the PTE enhancement ((**b**)—P183, (**d**)—P205, (**f**)—P282) under 1000 W m^−2^ solar simulator exposure. The performance of the pristine base fluid (ethylene glycol) is provided for comparison.

To ensure that the additional solar energy captured by the nanofluids from within the thermal spectral regions (see Section 3.1) was effectively converted into thermal energy within the PLDS working nanofluids, the temperature response and the corresponding PTE of the nanofluids were measured. The instantaneous change in the temperature of the working nanofluid ($\Delta T_{Fluid}$) in response to the loading concentration of the nanoparticles employed is presented in Figure 3 (a—P183, c—P205, e—P282); the thermal performance of the base fluid is also included. Although the heating rate and the stagnation temperature of the working nanofluid show an increase in the presence of a specific concentration of the nanoparticles, the enhanced thermal performance of the nanofluids becomes evident in the accompanying variations in the PTE enhancement. Consequently, by monitoring the variations in the PTE enhancement (Figure 3b—P183, d—P205, f—P282) throughout the heating cycle, a clearer understanding of the thermal behaviour of the nanofluids in response to the loading concentration of the nanoparticles can be ascertained.

As the nanofluids became exposed to the solar irradiance, the fluctuations in the temperature of the working nanofluid and the corresponding enhancement in the PTE increased by 5% (P205—Figure 3d—green star) to 170% (P183—Figure 3b—red square). Under the intense illumination of the artificial solar irradiance during this timeframe (0–10 min in Figure 3), the conduction processes, enabled by both the presence of the LDS materials and the nanoparticles, results in an upregulated thermal response of the working nanofluid. Some exceptions to the enhanced thermal behaviour were noted within this short term exposure window including pristine P282 (−6%, Figure 3f—red square), P282 at 1% *v*/*v* (−13%, Figure 3f—yellow triangle), P183 at 10% *v*/*v* (−3%, Figure 3b—green star), and P205 at 10% *v*/*v* (−6%, Figure 3d—green star) of nanoparticles, respectively.

After this initial period of adjustment, the fluctuations in the PTE enhancement began to decrease and stabilise due to the competing thermal interactions between the fluid and surrounding environment. Accordingly, the internal state of the nanofluid begins the process of re-orienting the individual constituents to accommodate the additional thermal energy via a combination of π–π stacking (LDS materials), particle agglomeration (nanoparticles), and interparticle interactions between the two species. The effect of these aggregation processes can be observed in the individual changes registered in the particle size data, after the total period of irradiance exposure had been sustained (Figure S3 post exposure—blue triangle). Furthermore, during the same timeframe (10–60 min in Figure 3) the PTE enhancement has increased for the different configurations of the nanofluid at specific loading concentrations of the nanoparticles. For example, over this 50-minute timeframe (10–60 min in Figure 3), for P183 at 1% *v*/*v* (Figure 3b—yellow triangle), P205 at 10% *v*/*v* (Figure 3d—green star), and P282 at 1% *v*/*v* (Figure 3f—yellow triangle) nanoparticles concetration the PTE enhancement increased by 6%, 8%, and 12%, respectively. These enhancements in the thermal behaviour of the nanofluid could be due to the concentration dependent nature of the aggregation processes which become energetically favourable within the nanofluid as the concentration of the nanoparticles is varied. Accordingly, the tendency of the nanofluids’ internal structure to accommodate the formation of extended particle chains, which are known to increase the thermal transport throughout the fluid, becomes concentration dependent [34]. The concentration of the nanoparticles which most significantly enhanced the PTE of the base fluid was determined to be 5% *v*/*v* for P183 (+12%), 1% *v*/*v* for P205 (+12%), and 10% *v*/*v* for P282 (+8%) PLDS nanofluids.

Although the enhancements observed in the PTE for the PLDS nanofluids do not look as significant as those reported in some of the purely nanomaterial-based approaches [21,31,35], one should consider that these nanofluids were not specifically designed to only act as thermal fluids for PT systems but, more specifically, to be used in hybrid PVT systems as well. Also, one should take into account the experimental factors that have to be considered when evaluating the PTE measurements as losses could occur as described in Supplementary Materials.

### 3.3. Performance of Concentrator Photovoltaic (C-PV) Solar Cell with PLDS Nanofluid Filters

**Figure 4:**
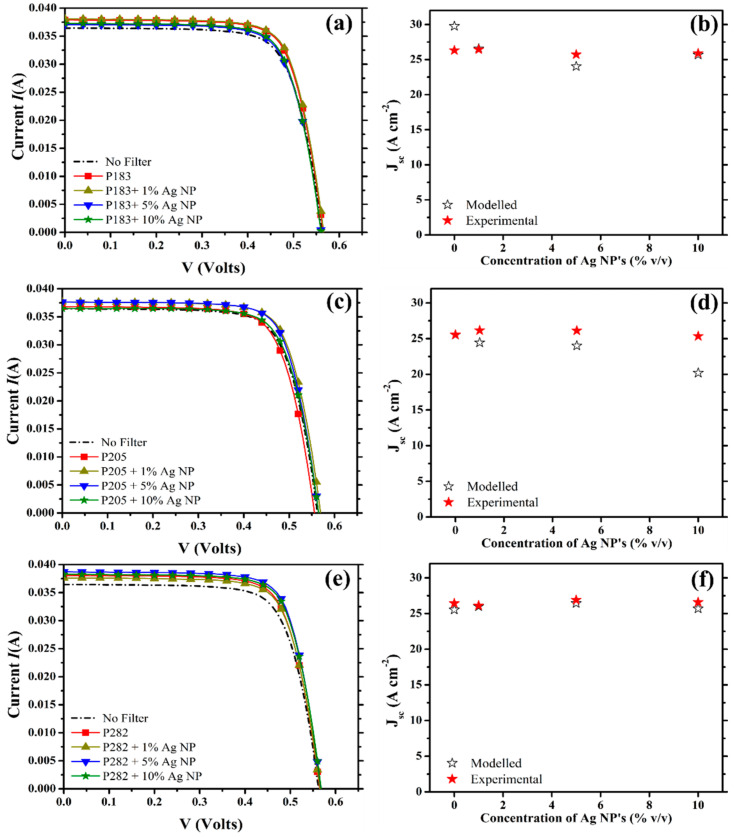
Current–voltage (I–V) response curve of the C-PV mc-Si cell when filtered by the (**a**) P183, (**c**) P205, and (**e**) P282 PLDS nanofluid filters employing different concentrations of the nanoparticles. The performance of the C-PV solar cell in the absence of any liquid filter (‘No Filter’) is also included. To be noted that only a small area (7 mm × 25 mm) of the mc-Si cell was exposed to the incoming irradiance during the experiment. The comparison between the impact of each nanofluid optical filter on the short circuit current density (J_SC_ in A cm^−2^) of the ‘filtered’ solar cell in response to an increased loading concentration of the nanoparticle ((**b**)—P183, (**d**)—205, (**f**)—P282) under the modelling (modelled—black unfilled stars) scenario and the experimental (experimental—red filled star) conditions is presented. The performances reported in each case reflect solar cell performances at a cell temperature 30 ± 0.2 °C.

The current–voltage (I–V) curves of the mc-Si C-PV cell response to the filtered spectral irradiance provided by the different PLDS nanofluid filters (outlined in Table 1) is shown in Figure 4 (P183—a, P205—c, P282—e) along with the electrical properties of the bare PV cell (Figure 4 ‘no filter’-dashed black line). In all instances the inclusion of only the organic luminescent materials contributed to an improvement of 0.22 A cm^−2^ (P205—Figure 2c—red square) to 1.52 A cm^−2^ (P282—Figure 2e—red square) in the current density J_SC_ produced by the cell. The enhancement in the electrical behaviour of the PV cell correlates closely with the intensity of the fluorescent emission of each species. Although P205 is more fluorescent than P183, P205 also sustains a 6% larger loss in the optical transparency across the electricity spectral region (Figure 2c). Hence, this additional loss in transparency could account for the fact that the relatively stronger emission of P205 failed to deliver an enhancement as significant as that offered by the P183 Imphen molecule. In contrast, the open circuit voltage (V_OC_) of the PV cell was found to decrease by 7 mV in the case of P205 (Figure 4c—red square) and increase by 2 mV for the remaining LDS materials. These subtle deviations in the V_OC_ of the mc-Si cell in response to a varying spectral irradiance is not unusual when performing an extensive series of electrical measurements like those encountered in the protocol outlined in 2.3.3 [57]. Therefore, the impact of the specific spectral modifications introduced into the transmittance and the accompanying fluorescence of the nanofluid (in the presence of the nanoparticles) was primarily ascertained through the associated changes registered in the J_SC_.

Through the inclusion of the nanoparticles, the enhancement in the J_SC_ becomes even more pronounced for each of the luminescent species at a specific loading concentration of the nanomaterial (Figure 4). At a nanoparticle concentration of 1% *v*/*v*, the J_SC_ was increased by 0.1 A cm^−2^ (P183—Figure 4a—yellow triangle) to 0.64 A cm^−2^ (P205—Figure 4c—yellow triangle), with the noticeable exception of P282 (Figure 4e—yellow triangle) which suffered a 0.32 A cm^−2^ decrease in the presence of the nanoparticles at this concentration. The decrease in the performance of P282 could be attributed to the 4% reduction in its fluorescence at this particular loading concentration or to the scattering contributions which originate from the increased number of large aggregates (diameter ~1000 nm) seen in the particle size data (Figure S3). As the concentration of nanoparticles is increased from 1% *v*/*v* (Figure 4—yellow triangle) to 10% *v*/*v* (Figure 4—green star), the effects of the concentration dependent spectroscopic changes discussed in Section 3.1 start to correlate with the variation of the conversion performance of the PV cell.

Furthermore, the concentration of the nanoparticles, which was deemed to optimise the fluorescent contributions for each particular luminophore (P183—0% *v*/*v*, P205—1% *v*/*v*, P282—5% *v*/*v*), corresponded to the concentration which maximised the PV collection efficiency. An exception is P183 as, in this case, the inclusion of even 1% *v*/*v* of the nanoparticles decreased the fluorescence of the PLDS nanofluid by 30% in comparison to its original value (Figure 2b—yellow triangle). In addition, at this loading of 1% *v*/*v* the optical transparency within the electricity spectral region was increased by 2%. This was most likely due to the interaction of the solar flux with the smaller sized aggregates (100–200 nm) which are revealed in the particle size data for the working nanofluid (Figure S3). Consequently, these results highlight the need to maintain the optical transparency of the PLDS nanofluids within the electricity spectral region. Any spectral deviations introduced in this sensitive region can substantially influence the PV cells behaviour and, consequently, that of the SBS-PVT system.

In addition, a comparison between the J_SC_ derived under the model scenario outlined in 2.4 (black unfilled stars) and the J_SC_ generated under the experimental conditions (red filled stars) is also provided in Figure 3 (P183—b, P205—e, P282—f) for further comparison. The divergence between the two measurements of the J_SC_ is indicative of the extent to which the fluorescent materials and the integration of the nanoparticles are compensating for the optical and reflective losses of the SBS-PVT system. Consequently, the concentration that maximises the difference between the two J_SC_ measurements is not the same as the concentration that maximises the performance of the PV element in the SBS-PVT system. Instead this particular configuration of the nanofluid represents the structure which best compensates for the losses within the collective SBS-PVT system and it can be considered to be the concentration which maximises the overall merit of the combined collection efficiency. Furthermore, the modelled PV cell performances do not include the contributions from the fluorescence, the plasmonic-enhanced fluorescence, or the reduced reflection losses than can arise as a result of the modifications of the refractive index of the liquid as an increasing number of nanoparticles are added. Therefore, the exhibited merit of the nanofluids internal structure to address some of the loss mechanisms, typically encountered in the hybridisation, becomes even more apparent. Consequently, the integration of the PLDS nanofluids in the SBS-PVT system not only enables the conversion efficiency of the PV component to be enhanced beyond that of the pristine mc-Si cell, but, simultaneously, promotes the heightened absorption of the nanofluid filters within the thermal spectral regions. This combination of the cooperative enhancement of both collection elements (thermal and electrical) makes this type of heat-transfer fluid even more appealing for the SBS-PVT applications.

### 3.4. Conversion Efficiency of SBS-PVT System and Merit Function with PLDS Nanofluids

**Figure 5:**
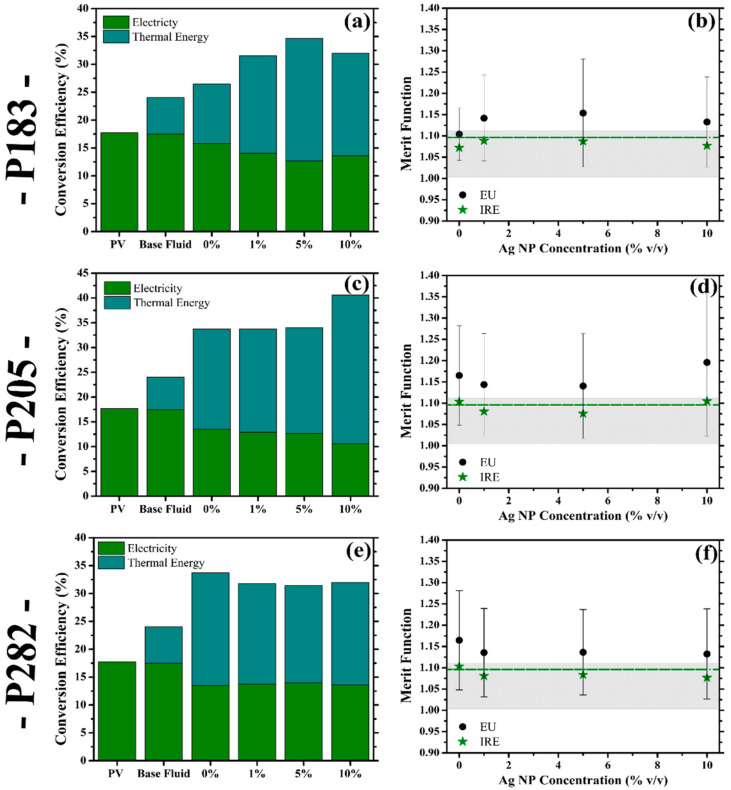
The combined conversion efficiency (%) of the spectral beam-splitting photovoltaic-thermal (SBS-PVT) system in response to the filtered spectral behaviour provided by the (**a**) P183, (**c**) P205, and (**e**) P282 PLDS nanolfuids. The changes in the performances of the individual electrical and thermal performances can also be observed. The electrical power of the unfiltered PV system is provided for comparison. The merit function of a C-PVT system incorporating mc-Si solar cells for each PLDS nanofluid filter ((**b**)—P183, (**d**)—P205, (**f**)—P282) according to the average worth factor derived across the European (EU—black circle) region, with the Republic of Ireland (IRE—geen star) economy being added for comparison. The filled region (light grey) and dashed line (green) represents the performance of the base fluid under the European conditions and the Republic of Ireland, respectively.

The capability of the PLDS nanofluids to independently enhance the thermal and the electrical conversion efficiency within the SBS-PVT system has been addressed in Section 3.2 and Section 3.3, respectively. Accordingly, the dynamic impact of the consolidated behaviour of the individual collection elements (PV and PT) on the energetic and economic merit of the SBS-PVT system was evaluated using the model and the merit function outlined in Section 2.4. The combined conversion efficiency achieved by hybridising the two collection systems as well as the corresponding changes in the efficiency of each sub-system (electricity—green, thermal energy—cyan) in response to the modifications in the internal structure of the nanofluid are presented in Figure 5 (a—P183, c—P205, e—P282). An enhancement in the combined conversion efficiency is achieved (Figure 5) irrespective of the configuration of the liquid optical filter integrated into the SBS-PVT system. Even the single component base fluid (pristine ethylene glycol) enables the capture of an additional 6.3% of the solar irradiance by virtue of its modest thermal collection efficiency (Figure 5a—base fluid-thermal energy). With the integration of the LDS materials into the base fluid the total collection efficiency of the SBS-PVT system is increased to 26.4% (P183—Figure 5a-0%) or 33.7% (P282—Figure 5c-0%). Considering the conversion efficiency of the standalone PV system stands at ~18% (Figure 5), the inclusion of even the purely organic species can be used to increase the energy efficiency of the hybridised system.

By including a fixed concentration of the nanoparticles in the PLDS nanofluids, the enhancement in the combined collection performance becomes further intensified. For example, in the case of P205 at 10% *v*/*v* (Figure 5c) and P183 at 5% *v*/*v* (Figure 5a) of the nanoparticles, the combined collection efficiency of the system was enhanced to 40.6% and 34.7%, respectively. Consequently, the presence of the nanoparticles in these instances contributed to a 20% and 31% enhancement in the conversion performance of the SBS-PVT system, respectively, when compared to their purely organic counterparts. Another interesting consequence to the inclusion of the nanoparticles in the PLDS nanofluid filters is the −3% (P183 5% *v*/*v*—Figure 5a) to +0.5% (P282 5% *v*/*v*—Figure 5e) variation in the efficiency of the PV element, as the collection efficiency of the thermal collector becomes considerably enhanced. Accordingly, the ability to maintain an electrical conversion efficiency close to that achieved with the base fluid may further enhance the economic merit of including the nanoparticles in the PLDS nanofluids. The capability to retain or enhance the capacity of the PV element to generate ‘clean electricity’ under the filtered irradiance of the PLDS nanofluid filters in the SBS-PVT system (Section 3.3) is the most important attribute considered during the evaluation of the merit of this novel class of nanofluids. The rationale behind this preferential weighted consideration of generating electricity over the alternative thermal energy in the hybrid SBS-PVT systems is due to the economic and energetic favourability of producing electricity in the global energy marketplace [31,48,49]. More importantly, the ratio of the deviation between the economic favourability of generating thermal energy as opposed to electricity (expressed as the worth factor in Equation (5)) is found in most developed economies to range from 0.8 (China) to 31 (Canada) [58,59,60,61]. Consequently, any variations in the conversion performance of the C-PV cell reported under the experimental conditions detailed in Section 3.3 will become amplified in the subsequent economic evaluation of the proposed modifications in the internal structure of the nanofluid. However, in some instances, a decrease in the combined conversion efficiency via the inclusion of the nanoparticles was noted. This is, most likely, the result of the PT and PV collector performances being evaluated based solely on the spectroscopic changes in the transmittance spectra of the PLDS nanofluids. Furthermore, the model (detailed in Section 2.4) from which these conversion performances were derived does not incorporate the fluorescent or plasmon-enhanced fluorescent contributions which have been shown to enhance the performance of the PV element in Section 3.3. Therefore, the conversion performances presented here reflect the PLDS nanofluids’ expected behaviour in the SBS-PVT system if they were non-fluorescent in nature. Yet, even under these circumstances, the spectroscopic changes registered through the inclusion of the LDS materials and, in some instances, the nanoparticles are contributing to an enhanced PVT performance.

In addition to the combined conversion performances of the PLDS nanofluids, the economic effect of their integration and the subsequent impact of the concentration of the nanoparticles in the economic context is also provided in Figure 5 (b—P183, d—P205, f—P282) by the inclusion of the merit function. The economic viability of the PLDS nanofluids (and correspondingly of the proposed modifications in the internal structure of the nanofluid) are presented under two different economic scenarios: the European region (Figure 5—black circle) and the Republic of Ireland (Figure 5—green star). In addition, the economic performance of the base fluid is also provided under both case studies (Europe—grey fill area, Republic of Ireland—dashed green line) for comparison. A merit function value of 1 implies that the merging of the two separate conversion systems resulted in a positive economic outcome for the resulting energy conversion system [31]. In this scenario, irrespective of the type of liquid optical filter selected, hybridising the PV and PT collection elements provided an economic merit through the improvements achieved in the collective conversion efficiency.

As the LDS materials are dispersed into the base fluid a further 8% (P183—Figure 5b) to 19% (P282—Figure 5f) enhancement in the economic merit of the additional energy captured is attained under the European scenario. Furthermore, through the inclusion of the nanoparticles at specific loading concentrations the economic merit of the additional energy captured by the SBS-PVT system can be further increased. For example, in the case of P183 at 5% *v*/*v* (Figure 5b), P205 at 10% *v*/*v* (Figure 5d) and P282 at 5% *v*/*v* (Figure 5f) of the nanoparticles’ concentration, respectively, the PLDS nanofluid delivered a 10%, 7% and 3% increase in the economic merit of the additional energy captured, when compared to their purely organic counterparts. Consequently, the inclusion of the nanoparticles in these configurations of the PLDS nanofluid are enhancing the economic merit of the SBS-PVT system through their advantageous spectroscopic properties. Remarkably, the concentration of the nanoparticles that was deemed to minimise the optical losses within the SBS-PVT system (inferred by the comparison of the different J_SC_ values in Figure 4) directly corresponds to the nanoparticle concentration which maximised the merit function of the PLDS nanofluids (P282—0% *v*/*v*, P205—10% *v*/*v*, P183—5% *v*/*v*). Consequently, the results highlight not only the premise that the inclusion of the nanoparticles can further compliment the optical and thermal behaviour of the LDS materials considered, but also their ability to control the dynamic between the thermal and electrical outputs of the SBS-PVT system.

In the merit function calculations presented in Figure 5, the European case study is inclusive of a large variety of different diverse economies, each with their own distinct energy markets and energy pricing. Consequently, the variation in the worth factor across the region inherently results in a variation in the economic viability of the PLDS nanofluids under the specific economic circumstances of each economic region considered. This variation in the economic performance of the PLDS nanofluids is expressed in the error bars associated with each individual loading concentration in Figure 5. Some of the modifications in the internal structure of the nanofluid can lead to a decrease in the economic merit of the additional energy captured as a result of the hybridisation. Some examples of the European countries where this tended to be the case were those where the worth factor value exceeded ~3.5 (e.g., Germany, Belgium, the UK, Croatia, and Turkey). Interestingly, the capability of the presence of the nanoparticles in the PLDS nanofluids to maintain a marginal drop in the efficiency of the PV component, whilst offering an increased thermal efficiency, seems to suggest that this type of fluid design will perform better under these economic conditions. However, these considerations could imply that the PLDS nanofluids could be more advantegous for countries where the energy infrastructure supports the additional thermal and electrical energy that is being captured by the hybridised optical filters.

## 4. Conclusions

In this study a series of fluorescent imidazo-phenanthroline molecules were combined with Ag nanoparticles to develop plasmon-enhanced luminescent down-shifting nanofluids for SBS-PVT applications. The fluorescent molecules were dispersed in ethylene glycol and the resulting nanofluids’ optical transmittance, fluorescence, and thermal response to the normal incidence of the solar irradiance were measured as a function of the concentration of the nanomaterial added. The resulting nanofluids exhibited a significantly increased absorption in the ultraviolet and the infrared solar spectral regions (which are typically designated for the capture of thermal energy) as compared with the pristine base fluid. These complimentary absorption properties of the composite nanofluids attributed an 8–12% enhancement in the photothermal conversion efficiency of the working nanofluid. Furthermore, when compared to the performance of the base fluid containing only the organic molecules, the inclusion of the Ag nanoparticles leads to enhancements in the thermal performance with up to 20% for PT systems and up to 11% for the PVT configurations. The controlled capability of the composite nanofluids to selectively modulate the solar spectrum was further augmented by the nanoparticles’ presence, whose addition under specific loading concentrations served to modify the fluorescence and scattering contributions of the liquid optical filters. The unique features of the nanoparticle ensemble in some instances contributed up to a 13% enhancement in the intensity of the emission spectra of the down-shifting species, which were cooperatively located within the peak spectral responsivity of the silicon solar cell.

In addition, indoor tests were conducted to investigate the effect of the composite nanofluids on the electrical performance of a mc-Si C-PV cell. In the cases where the radiative transitions of the imidazo-phenanthroline molecules were enhanced by the presence of the Ag nanoparticles, a 0.10 A cm^−2^ to 0.64 A cm^−2^ increase in the short circuit current density of the mc-Si PV cell was achieved. Under these circumstances the combination of the two materials increased the exergetic performance of the SBS-PVT system by enabling more electricity to be generated. In all other configurations tested, the presence of the nanofluid resulted in an enhanced thermal performance (1–11%), whilst only suffering a modest reduction (0.5–3%) in the electrical conversion efficiency when compared with the standalone PV system.

The results demonstrate that plasmon-enhanced luminescent down-shifting nanofluids can be exploited within PVT systems in order to maximise the solar energy conversion, in general, and the electricity generated, in particular. Thus, the unique combination of the advantageous properties exhibited by the composite nanofluids enabled the combined collection efficiency of the PVT system to be increased by 8% to 23%, while maintaining an electrical conversion efficiency close to that achieved with the single component base fluid. However, further increases in the performance of the hybridised collection system are envisioned to be attainable by optimising the concentration of the fluorophores employed, the orientation of the collector housing, the flow conditions of the fluid, and the architecture of the thermal collection unit etc. The energetic merit of including the nanoparticles in the composite filter’s structure was found to be influenced by the onset of concentration-dependent processes (π–π stacking, nanoparticle agglomeration, and interparticle interactions between the two species) which resulted in the reconfiguration of the internal state of the filter.

The ability of the proposed composite nanofluids to convert more effectively the solar irradiance into electricity and thermal energy within an SBS-PVT system was also economically evaluated using a merit function. In fact, in designing this novel nanofluid, along with considering its optical properties that could enhance solar energy conversion, the production cost of the fluorescent additives was also considered. Thus, we have estimated that the cost of their inclusion to reach the efficiencies reported in this study, equates to an additional 5–6 cents per litre of ethylene glycol. The strong absorption contribution in the targeted thermal spectral regions, arising from the inclusion of the organic fluorescent species within ethylene glycol, resulted in an 8% to 19% increase in the economic value of the energy captured. Furthermore, the additional 3–10% increase in the economic value of the energy captured by combining the two materials, highlights the promise of the nanomaterial to further compliment the optical and thermal behaviour of the down-shifting materials considered. Additionally, the capability to retain or enhance the capacity of the PV element to generate ‘clean electricity’ under the filtered irradiance of the composite nanofluid filters in the SBS-PVT system represents the most important attribute highlighted during the evaluation of the merit of this novel class of nanofluids. Also, it should be noted that the results obtained using these first-generation nanofluids could be further optimized by investigating their thermal properties dependence on concentration, molecular structure of the LDS, and the effect of irradiation angle etc. Also, like for any other nanofluid, further studies on ageing, toxicity and environmental impacts will have to be performed during the future developmental cycle of these materials.

The merit function reflects, in a weighted manner, the preference given to the generation of electricity over the alternative thermal energy in the hybrid SBS-PVT systems. This preference will be determined by the economic and the energetic favourability of producing electricity in the global and/or regional energy marketplace. However, the implementation of PVT systems will depend on the worth factor which will be characteristic for each geographical region. Currently, most of the electrical energy produced by PV systems is being used for spatial heating and the production of hot water within the residential and industrial sectors. Consequently, through implementing a small-scale PVT system a proportion of this thermal capacity could be capitalised upon at a localised level by each individual user. Moreover, implementing a SBS-PVT system would require minimal modification to existing PV panel architecture as the technology could be retrofitted to existing systems already in operation, resulting in a much smaller initial capital investment. Consequently, it is expected that for localised regions where PV systems are economical viable or in remote areas or regions where the thermal energy (hot water for heating or household consumption) is produced using electricity SBS-PVT systems could become an advantageous alternative. 

## Figures and Tables

**Table 1:** The molecular structure of the materials used in this study as PLDS liquid optical filters.

Abbreviation	Chemical Formula	Name	Molecular Weight (g mol^−1^)
P183	C_32_H_18_N_8_	1,4-bis(1*H*-imidazo[4,5-*f*][1,10]phenanthroline-2-yl)benzene	514.55
P205	C_20_H_11_N_5_	4-(1*H*-imidazo[4,5-*f*][1,10]phenanthroline-2-yl)benzonitrile	321.44
P282	C_29_H_16_N_4_	2-(pyren-1-yl)-1*H*-imidazo[4,5-*f*][1,10]phenanthroline	420.42

## Supplementary Materials

The following are available online at https://www.mdpi.com/2079-4991/10/6/1201/s1: Figure S1: Schematic diagram of the SBS-PVT configuration, Figure S2: The calculated transmittance spectra of the PLDS nanofluid filters, Figure S3: The variation in the internal structure (particle size-distribution) of the different types of the PLDS nanofluid in response to the increased concentration of the silver nanoparticles, Figure S4: The instantaneous change in the surface temperature of the C-PV mc-Si cell ($\Delta T_{PV\;Cell}$) under the different filtered spectral irradiances provided by the PLDS nanofluids.
