# Supplementary material for: Nanofluid Development Using Silver Nanoparticles and Organic-Luminescent Molecules for Solar-Thermal and Hybrid Photovoltaic-Thermal Applications
Source: Nanomaterials (Basel). 2020 Jun 19;10(6):1201. doi: 10.3390/nano10061201 (PMC7353118; doi:10.3390/nano10061201)
Supplement: Supplementary file 1 [file nanomaterials-10-01201-s001.pdf]

nanomaterials

Supplemental Material

Nanofluid Development Using Silver Nanoparticles and Organic-Luminescent Molecules for Solar-Thermal and Hybrid Photovoltaic-Thermal Applications

James Walshe [1,2], Pauraic Mc Carron [3,4], Conor McLoughlin [1], Sarah McCormack [5], John Doran [1,2] and George Amarandei [1,*]

1 School of Physics & Clinical & Optometric Sciences, Technological University Dublin, City Campus, Kevin Street, Dublin, D08 NF82, Ireland; james.walshe@tudublin.ie (J.W.); c15442598@mytudublin.ie (C.M.); john.doran@tudublin.ie (J.D.)
2 Dublin Energy Lab, Technological University Dublin, City Campus, Dublin, D08 NF82, Ireland
3 School of Science & Computing, Technological University Dublin, Tallaght Campus, Dublin, D08 NF82, Ireland; pauraic.mccarron@tudublin.ie (P.M.C.)
4 The Centre for Biomimetic & Therapeutic Research, Technological University Dublin, City Campus, Dublin, D08 NF82, Ireland
5 Department of Civil, Structural & Environmental Engineering, Trinity College Dublin, Dublin, D08 NF82, Ireland; mccorms1@tcd.ie (S.M.)
* Correspondence: george.amarandei@tudublin.ie (G.A.)

Received: 20 May 2020; Accepted: 17 June 2020; Published: 19 June 2020

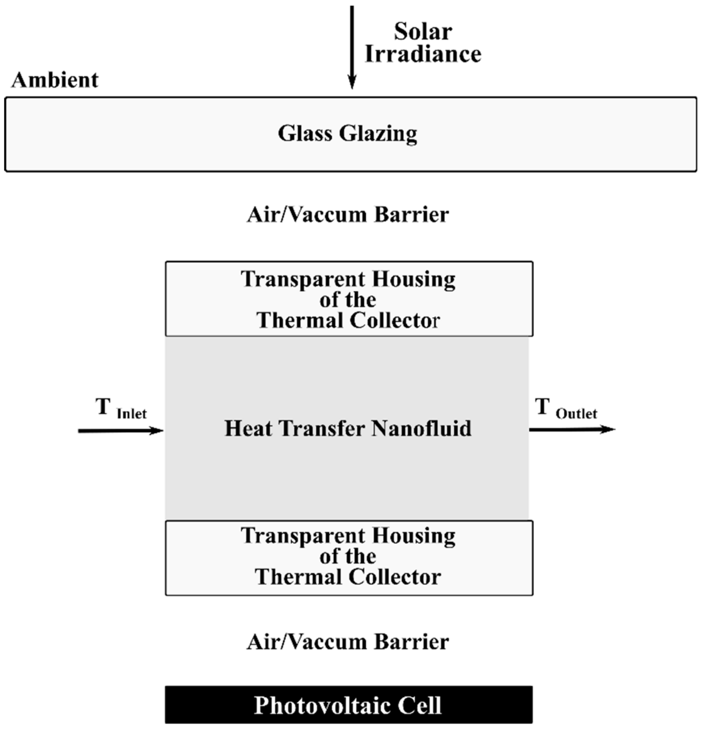

Figure S1. Schematic diagram of the spectral beam-splitting photovoltaic-thermal (SBS-PVT) configuration discussed in section 2.3.3 and 2.3.4 in relation to the electrical characterization of the mc-Si photovoltaic (PV) cell and the photothermal conversion efficiency of the thermal collector, in response to the internal configuration of the liquid optical filter, respectively.

Modelled Transmittance Spectra of Plasmonic-Enhanced Luminescent Down-Shifting (PLDS) Nanofluids

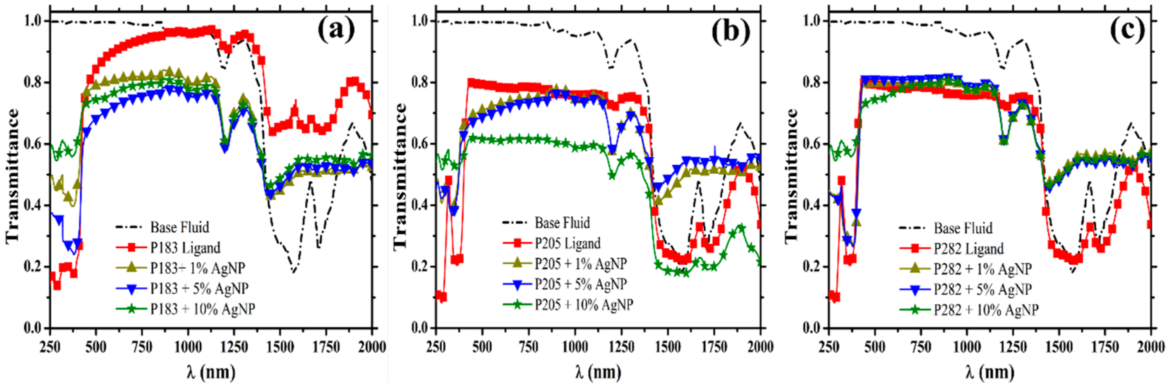

Figure S2. The calculated transmittance spectra of the (**a**) P183, (**b**) P205, and (**c**) P282 plasmonic-enhanced luminescent down-shifting (PLDS) nanofluid filters in response to the varying concentration of silver nanoparticles, with the optical properties of the base fluid (ethylene glycol) included.

Particle Size of PLDS nanofluids

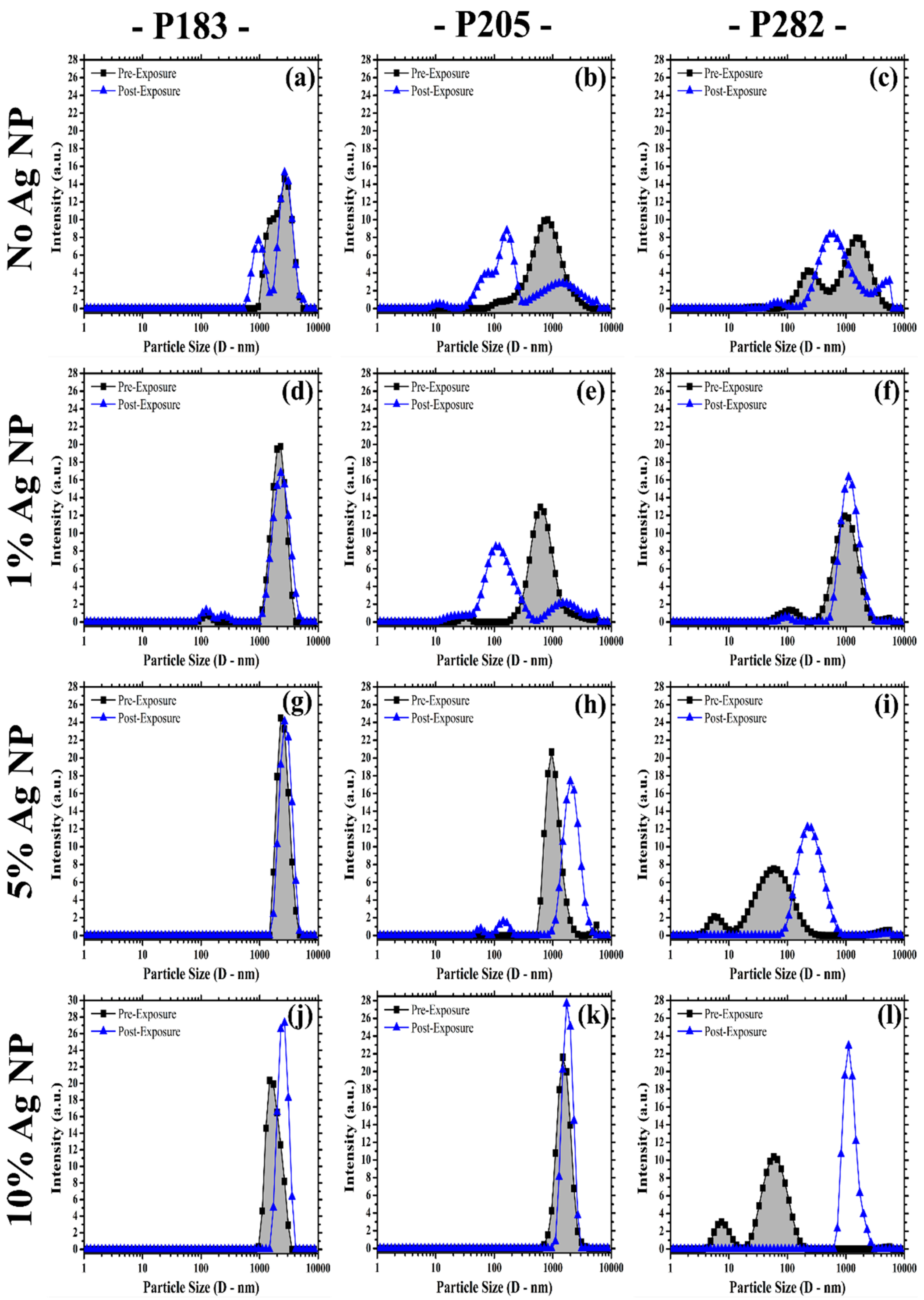

Figure S3. The variation in the internal structure (particle size-distribution) of the different types of the PLDS nanofluid in response to the increased concentration of the silver nanoparticles. The particle size-distribution pre-exposure (blue triangle) and post-exposure (black square) to the solar irradiance is provided to show any changes in the internal structure of the nanofluid which could have resulted from the aggregation processes.

Solar Cell Temperature under the Filtered Scenarios

One should consider the losses associated with the experimental parameters when evaluating the photothermal conversion efficiency (PTE) measurements. Such losses can occur through the possible leakage of thermal energy through the window created in the photovoltaic/thermal (PVT) sample holder to allow the filtered irradiance to strike the concentrator photovoltaic (C-PV) cell. Certainly, the possibility of this thermal energy escaping through the non-insulated window layer would assist in explaining the behaviour of the temperature of the C-PV solar cell ($\Delta \boldsymbol{T}_{\boldsymbol{PV\ Cell}}$) when the various plasmonic-enhanced luminescent down-shifting (PLDS) nanofluid filters were employed (Figure S3). For some of the configurations of the PLDS nanofluid filter, the $\Delta \boldsymbol{T}_{\boldsymbol{PV\ Cell}}$ is larger than the response of the unfiltered exposed C-PV solar cell. While the increase in the solar cell temperature only amounted from 0.7 °C (P205—Figure S3b—yellow triangle) to 1.3 °C (P282—Figure S3c—inverted blue triangle), even these small losses from the collection system (when included in the PTE calculations) would have pushed the enhancement in the PTE to 20% (P183 at 0% v/v) to 27% (P282 at 5% v/v). However, one might speculate that the contributions from the plasmon-enhanced fluorescence of the luminescent down-shifting (LDS) materials is in fact the origin of the fluctuations in the solar cell temperature. If this would have been the case, then the largest increase in $\Delta \boldsymbol{T}_{\boldsymbol{PV\ Cell}}$ would directly correlate with the most intense fluorescence attributed to the presence of the nanoparticles, which would be in order of decreasing magnitude P282 (5% v/v—Figure S3c-blue inverted triangle), P205 (1% v/v—Figure S3.b—yellow triangle), and P183 (0% v/v—Figure S3a-red square). Also, considering that P282 is three times more fluorescent than P183 in the purely organic working nanofluids, it would be expected that P282 would have sustained a much larger increase in cell temperature, if fluorescence was in fact the origin of the temperature variations. Furthermore, in the transmittance spectra of the PLDS nanofluid configurations (see section 3.1.) in which the apparent 'thermal loss' is maximised (Figure S3) an intensified absorption within the thermal spectral regions of the solar spectrum is also noted (see section 3.1). Therefore, the importance of ensuring that the thermal collection unit is adequately insulated in the SBS-PVT system is of primary importance if the nanofluids' thermal collection efficiency is to be maximised.

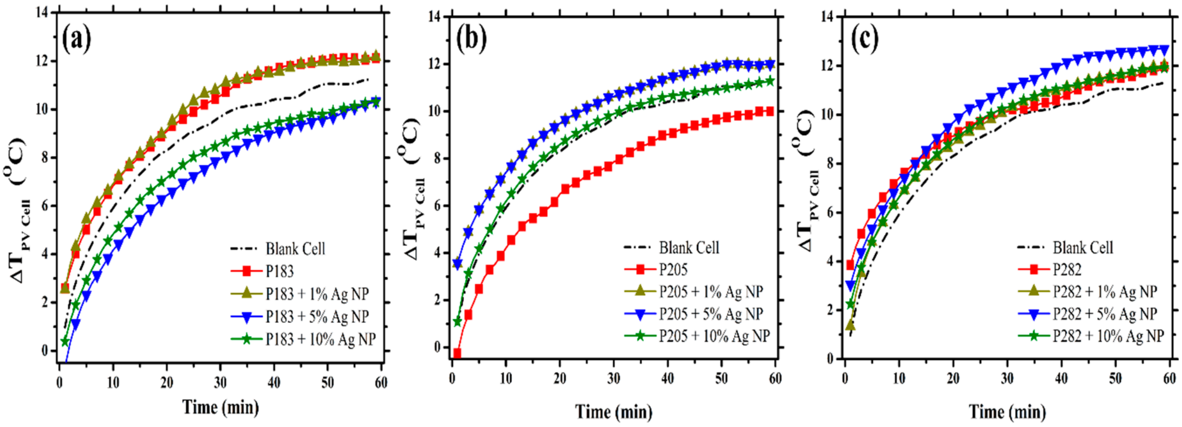

Figure S4. The instantaneous change in the surface temperature of the concentrator photovoltaic (C-PV) mc-Si cell ($\Delta T_{PV\ Cell}$) under the different filtered spectral irradiances provided by the (**a**) P183, (**b**) P205, and (**c**) 282 PLDS nanofluids. The thermal response of the unfiltered standalone PV system (blank cell – dashed black line) is also included.

© 2020 by the authors. Licensee MDPI, Basel, Switzerland. This article is an open access article distributed under the terms and conditions of the Creative Commons Attribution (CC BY) license (http://creativecommons.org/licenses/by/4.0/).
